# Skin cancer prevention in the Polish population during the COVID-19 pandemic

**DOI:** 10.3389/fpubh.2025.1452043

**Published:** 2025-06-20

**Authors:** Izabela Jęśkowiak-Kossakowska, Jacek Calik, Adam Szeląg, Benita Wiatrak

**Affiliations:** ^1^Department of Pharmacology, Faculty of Medicine, Wroclaw Medical University, Wrocław, Poland; ^2^Department of Clinical Oncology, Wroclaw Medical University, Wrocław, Poland; ^3^Old Town Clinic, Wrocław, Poland

**Keywords:** melanoma, skin cancer, sun exposure, photosensitive medicines, COVID-19 pandemic, sun protection

## Abstract

**Introduction:**

In addition to chronic skin inflammation, exposure to ultraviolet radiation (UVR) from sunlight is one of the most important factors predisposing to skin cancer. The aim of the study was to determine the occurrence of significant risk factors for skin cancer and to assess the methods of skin cancer prevention used in the Polish population during the COVID-19 pandemic.

**Methods:**

An anonymous survey was conducted between December 2021 and December 2022. 651 respondents took part in the study, including 86 respondents (13.2%) suffering from skin cancer.

**Results:**

It was found that statistically significantly more often respondents with atopic dermatitis (*p* < 0.001), rosacea (*p* = 0.002), alopecia areata (*p* < 0.001), diabetes mellitus (*p* < 0.001), hypertension (*p* < 0.001), rheumatoid arthritis (*p* < 0.001) or Crohn's disease (*p* < 0.001) had skin cancer. Moreover, participants using medicines that could cause photodermatoses were more likely to suffer from skin cancer (*p* < 0.001), sunburn (*p* = 0.005) and have moles removed (*p* = 0.014) as well as more likely to have Sutton's nevus (*p* = 0.034) and Becker's nevus (*p* < 0.001). Skin cancer was diagnosed more often in participants with Celtic complexion (*p* < 0.001) and respondents with Celtic complexion were much more likely to have family members diagnosed with skin cancer (*p* = 0.014). The incidence of skin cancer (*p* < 0.001), Sutton's nevi (*p* = 0.007), Becker's nevi (*p* = 0.029) and mole removal (*p* < 0.001) increased with participant age. Women (*p* < 0.001) and respondents with Celtic and Northern European skin types (*p* < 0.001) most often choose creams with SPF50, but respondents with Southern European skin were the least likely to declare sunburn (*p* < 0.001). On sunny days more often, men (*p* < 0.001) and older respondents (*p* = 0.040) wear headgear and women wear sunglasses (*p* = 0.018). Women also supplemented vitamin D more often (*p* < 0.001). More women (*p* < 0.001) and younger respondents (*p* < 0.001) know the ABCDE method, which allows for quick identification of potential melanoma.

**Conclusions:**

Regular examination of moles, in addition to adequate skin protection against UVR, is an important element of skin cancer prevention, especially in people with fair skin, those suffering from inflammatory skin diseases and diabetes as well as taking medications with photosensitizing properties.

## 1 Introduction

Aging and exposure to ultraviolet radiation (UVR) influence the increasing presence of three main forms of skin cancer: basal cell carcinoma (BCC), squamous cell carcinoma (SCC), and cutaneous malignant melanoma (MM) (1–4). These types of skin cancer are the most common among Caucasians. The occurrence of SCC is associated with chronic exposure to UVR, and MM is associated with periodic excessive sun exposure and sunburn in childhood (5–7). In particular, MM and BCC are more common in young women and older men (8). Younger women's skin is much more sensitive to sunlight than that of people over 50 (9). Also, children may be more susceptible to skin damage from UVR because their biological defense systems are not fully developed (10). High birth weight and high exposure to UVR early in life may be independent, significant risk factors for developing MM before the age of 30 (11).

Melanomas are responsible for 80% of skin cancer deaths (12–15). Advanced MM is associated with poor survival of 6–7 months without treatment (16). The incidence of MM varies geographically. The highest incidence of MM in the world occurs in Australia and New Zealand (17). Melanoma is more common in people with blue or green eyes, red or blonde hair, people who react to light by sunburn rather than tanning, and who have sunspots (18). People with fair skin are more susceptible to initiating cancer processes, especially MM, under the influence of UVR compared to people with dark skin (19–23). Results from the prospective cohort QSkin Sun and Health Study showed that country of birth and sunburn in childhood or adolescence are factors that significantly increase the risk of MM (24). An increased trend in the incidence of MM since 1975 has been observed in both Caucasian women and men. During the same period, men experienced higher MM morbidity and mortality compared to women (25).

According to the Central Statistical Office (GUS) data, in Poland the incidence of MM and other skin cancers in 2018 was 46.5 (per 100,000 population), and in 2019 it was 46.8 (per 100,000 population). Moreover, women in Poland suffer from skin cancer more often than men. In the unusual pandemic year of 2020, 124,000 new cases of malignant tumors were recorded in Poland. It was 14.7% less compared to 2019. Incidence rate per 100,000 population amounted to 372.3 cases, 61.8 less than in the previous year. In 2020, fewer cases of all types of cancer were registered, but the structure of cases, considered on a national scale, was similar to 2019. In 2020, the incidence rates in the case of melanoma and others skin cancers were 36.2–10.5 cases less than in the previous year [source: https://stat.gov.pl/obszary-tematyczne/zdrowie/zdrowie/zdrowie-i-ochrona-zdrowia-w-2021-roku,1,12.html (accessed on 08 June 2024)]. The COVID-19 pandemic has caused major disruptions in the delivery and use of healthcare services. Across the world, healthcare systems have seen reductions in patient visits and diagnostic tests (26, 27). The number of MM cases diagnosed annually has decreased by ~31.37 and 23.75% in the first and second year after the pandemic, respectively, compared to pre-pandemic numbers (28). The coronavirus pandemic has disrupted the entire healthcare system on a large scale. The pandemic had the greatest impact on screening tests due to the lockdown in April–June 2020, which resulted in a decrease in the number of patients who were issued oncology diagnosis and treatment (DiLO) cards and were treated for cancer. Due to the pandemic, access to treatment has been significantly hampered, creating health debt that the healthcare system will now have to deal with (29–32).

It is worth emphasizing that the pandemic also had a positive impact on society by raising awareness of the importance of health and thus building awareness of the need to perform preventive tests and vaccinations (33, 34). At the same time, due to the COVID-19 pandemic health issues have become a priority demanding more attention and influencing patient expectations, such as increased financing for access to a wider range of tests in primary healthcare.

The aim of the study was to determine the occurrence of significant risk factors for skin cancer and to assess the methods of skin cancer prevention used in the Polish population during the COVID-19 pandemic, along with the preparation of proposals for recommendations for Polish residents who are particularly predisposed to skin cancer.

## 2 Materials and methods

### 2.1 Study design, population and sampling

An anonymous survey was conducted in the form of an electronic survey. In December 2021 electronic surveys were sent to employees and students of the Medical University of Wrocław, and in January 2022 to the District Pharmaceutical Chambers and District Medical Chambers. It is difficult to determine to what extent all counties' medical and pharmaceutical chambers conducted the survey. In February 2022, electronic surveys were posted on various forums dealing with health and cancer. Subsequently, an anonymous electronic survey was conducted among patients of the Old Town Clinic in Wrocław and the Beata Kostrzewa massage and rehabilitation office in Bolesławiec in the period from March to December 2022. Google Questionnaire provides features for designing online questionnaires and surveys for enterprises, research institutions and private individuals. The survey consisted of 28 questions. A total of 651 participants completed the survey, including 86 (13.2%) respondents belonging to the group of people suffering from skin cancer. The study was approved by Bioethics Committee of the Medical University of Wrocław (KB1039/2021).

### 2.2 Content of questionnaire

**Figure 1:**
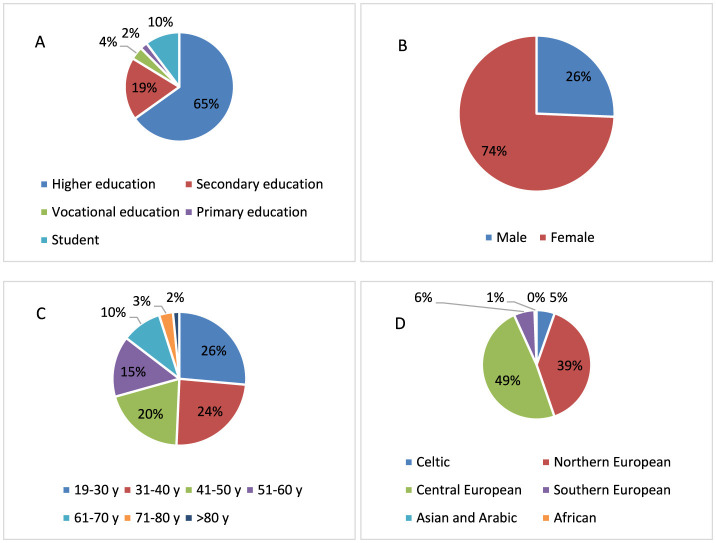
The characteristics of the study group: **(A)** education, **(B)** sex, **(C)** age, and **(D)** type of complexion.

The content of the questionnaire included [A] social characteristics, such as education (higher, secondary, doctoral, student, professional), gender (female, male), age (divided into groups 19–30, 31–40, 41–50, 51–60, 61–70, 71–80, and over 81 years) [B] type of complexion: [1B] Celtic—very fair skin, light pink or white, blonde or red hair, light eye color (blue, gray or light green), does not tan, gets sunburn immediately; [2B] Northern European—pale skin, red, light to dark blond and light brown hair, blue, hazel or green eye color, minimal tan, high tendency to burn; [3B] Central European—light skin in warm tones (beige and gold), hair from dark blonde to dark deep brown, eye color gray, hazel, green or brown, always tans, slight tendency to burn; [4B] Southern European—swarthy skin light brown or olive brown, hair dark brown or black, eyes intensely brown, always and easily tans, almost never burns; [5B] Asian and Arabic—naturally dark olive skin, black hair, dark eyes, usually brown or black, tans well, does not tend to burn, [6B] African—medium brown to dark brown skin, black hair, eye color dark brown or black, no burns—Graphic summary presented in Figure 1; [C] factors increasing the risk of skin cancer: [1C] skin disease; [2C] using sunscreen creams with sun protection factor (SPF) filter while staying in the sun; [3C] using a solarium; [4C] using moisturizing cosmetics after a long stay in the sun or tanning; [5C] sunburn in the past; [6C] wearing a headgear on sunny/hot days; [7C] wearing sunglasses on sunny/hot days; [8C] check-ups with a dermatologist; [9C] smoking; [10C] vitamin D supplementation; [11C] diagnosis of skin cancer in a close relative (parents, siblings, and grandparents); [12C] mole removal procedure in the past; [13C] presence of nervus: [13C.1] Sutton's, [13C.2] Becker's, [13C.3] blue; [D] knowledge about the ABCDE formula for observing moles; [E] knowledge about drugs causing photodermatoses; [F] regular use of medications that may cause photodermatoses; [G] diagnosed skin cancer: [1G] type of skin cancer; [2G] how many years have passed since the diagnosis of skin cancer; [3G] how many years have passed since the end of skin cancer treatment; [4G] type of treatment used.

### 2.3 Statistical analysis

Statistical analyses were performed with Statistica v13.0. Pearson's chi-square test was used to compare the differences between the different subgroups.

## 3 Results

### 3.1 Study sample characteristics

Figure 1 is a graphic representation of the study group. The majority of participants in this anonymous survey were respondents with higher education (65.0%), respondents with secondary education accounted for 19.0%, and 10.0% were students (Figure 1A). Due to the small number of respondents with vocational education and primary education, the inclusion criteria in the study of the impact of education were higher education, secondary education and students who were qualified for secondary education. The exclusion criteria were primary education and vocational education. The majority of respondents were women (74.0%; Figure 1B). The age of the respondents was evenly distributed (Figure 1C). The majority of respondents had the Central European skin type (52.0%) and, to a lesser extent, Northern European skin type (37.0%; Figure 1D).

The inclusion criterion for the study was the respondent's age of 19 years and over. In the case of the analysis of the impact of education on sun protection factors, the exclusion criteria were primary education and vocational education. Two educational groups were analyzed: higher education and secondary education, which also included students. From the surveyed group of respondents [651], a group of patients diagnosed with skin cancer was identified [86].

**Figure 2:**
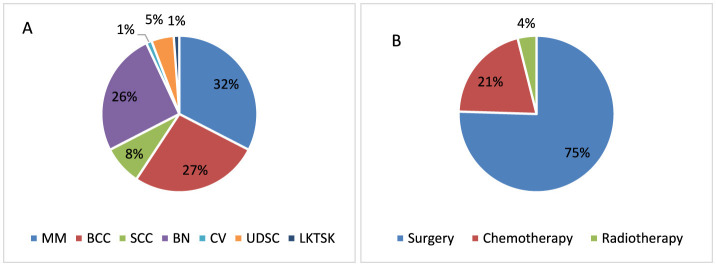
Distribution of skin cancer types **(A)** and treatment methods used **(B)** among respondents in the study group.

The inclusion criterion for the group of respondents suffering from skin cancer was the presence of MM—[28], BCC—[23], SCC—[7], carcinoma verrucosum (CV)—[1], benign cancer (BN)—[22], patients undergoing diagnosis of skin cancer (UDSC)—[4], patients who have lack of knowledge about the type of skin cancer, but they have been diagnosed with lack of knowledge about the type of skin cancer (LKTSK)—[1]. Three methods of skin cancer treatment, i.e., surgery [77], chemotherapy [21], and radiotherapy [4], were used in this group of skin cancer respondents. In the study group, the dominant treatment method was surgical removal of cancerous skin lesions. Figure 2 is a graphic representation of the distribution of skin cancer types among respondents (Figure 2A) and the treatment methods used (Figure 2B).

### 3.2 Factors increasing the risk of skin cancer

#### 3.2.1 Skin diseases

**Figure 3:**
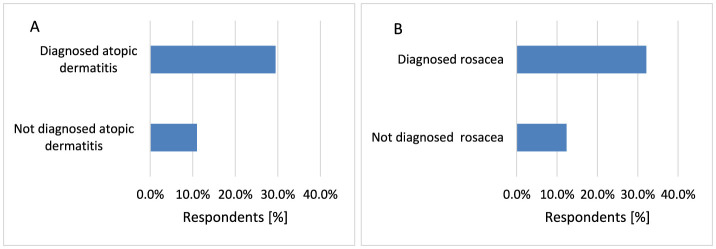
Respondents suffering from AD [**(A)** *p* < 0.001] and Ros [**(B)** *p* = 0.002] were diagnosed with skin cancer significantly more often.

The chronic inflammation, which occurs in inflammatory skin disease, can damage DNA and potentially alter the risk of mutations that lead to cancer (35); the co-occurrence of other skin diseases may increase or decrease the risk of skin cancer. Recent data suggest a decreased risk of MM in people with atopic dermatitis (AD), but an increased risk of other skin cancers, especially non-melanoma skin cancer (NMSC), including BCC and SCC (36, 37). The study respondents suffering from AD were also diagnosed with skin cancer more often (*p* < 0.001, 29.5 vs. 11.0%; Figure 3A). AD is a chronic recurrent inflammatory skin disease associated with epithelial, immune, and environmental factors, which is characterized by activation of the type-2-mediated immune response in the skin breakdown of the skin barrier, and intense itching (38).

Rosacea (Ros) is the most common inflammatory skin condition among adult inhabitants of Northern European with light-skinned heritage (39), which is characterized by facial erythema, pustule papules, and teleangiectasia. UVR from natural sunlight can worsen Ros symptoms (40). Ros and cancer are believed to be linked by the common occurrence of inflammatory disorders and immune response disorders (32). Participants with Ros were significantly more likely to suffer from skin cancer (*p* = 0.002, 32.1 vs. 12.4%; Figure 3B).

**Figure 4:**
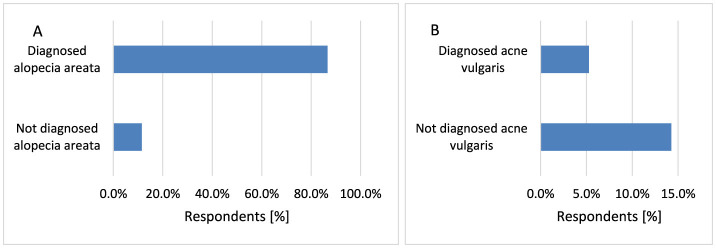
The respondents suffering from AA were more likely to have been diagnosed with skin cancer [**(A)** *p* < 0.001], whereas those suffering from AV were more likely not to have skin cancer [**(B)** *p* = 0.029].

Alopecia areata (AA) is a chronic, inflammatory, common autoimmune disease characterized by non-scarring hair loss that affects all ages, both sexes, and all skin types (41). However, current data show that individuals of non-Caucasian origin are more prone to disease development (42). Psychological stress has been proposed as an external factor that contributes to the development of AA (43). However, histological examination revealed inflammatory cell infiltrates around the bulbar region of hair follicles in patients with AA (44). The conducted research presented that, similarly to survey participants suffering from AD and Ros, respondents diagnosed with AA are more likely to suffer from skin cancer (*p* < 0.001, 86.7 vs. 11.5%; Figure 4A).

Acne vulgaris (AV) is common among young people (45) and reflects hormonal imbalance and may be a key component of many systemic diseases. It was hypothesized that the diagnosis of AV in adolescents may predict subsequent cancer risk (46). However, respondents in this study with AV were significantly more likely not to suffer from diagnosed skin cancer (*p* = 0.029, 5.3 vs. 14.3%; Figure 4B).

#### 3.2.2 Other chronic diseases

**Figure 5:**
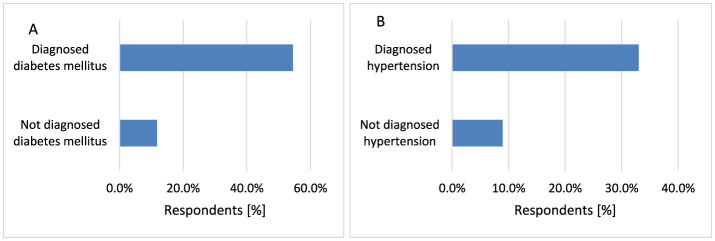
Respondents suffering from DM [**(A)** *p* < 0.001] and hypertension [**(B)** *p* < 0.001] were diagnosed with skin cancer significantly more often.

Diabetes mellitus (DM) is defined as a chronic, systemic condition characterized by hyperglycemia, leading to severe complications, including neuropathy. There is a significant portion of diabetic patients experiencing skin-related complications. The issues arising from DM are largely attributed to chronic hyperglycemia and elevated fatty acid levels, with oxidative stress playing a crucial role in the patho-mechanism of this disease (47). The state of oxidative stress in diabetes, with consequential DNA damage, is also considered responsible for the transformation of oncogenes and development of cancers (48). This study showed that respondents diagnosed with diabetes have an increased risk of skin cancer (*p* < 0.001, 54.5 vs. 11.8%; Figure 5A).

Hypertension is defined as high systolic and/or diastolic blood pressure (49). Several anti-hypertensive drugs are photosensitizing and may therefore act as co-carcinogens with UVR, which can increase the risk of skin cancer (50, 51). Some studies indicated that the use of hydrochlorothiazide was associated with an increased risk of SCC but no association was observed for BCC or melanoma (52). This study showed that the presence of hypertension increases the risk of skin cancer (*p* < 0.001, 33.0 vs. 9.0%; Figure 5B).

**Figure 6:**
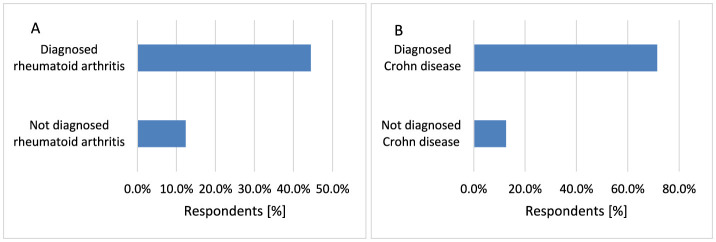
Respondents suffering from RA [**(A)** *p* < 0.001] and CD [**(B)** *p* < 0.001] were diagnosed with skin cancer significantly more often.

Rheumatoid arthritis (RA) is a chronic inflammatory condition with joint swelling, pain and stiffness (53). This study indicated that suffering from RA increases the risk of skin cancer (*p* < 0.001, 44.4 vs. 12.3%; Figure 6A). A Swedish study showed the risk of NMSC may be increased in patients with RA (54). An increased risk of MM in inflammatory bowel disease, including Crohn disease (CD) has been reported (55, 56). Also, the greatest risk of NMSC was indicated for CD patients (57). Treatment with thiopurine for more than 5 years was associated with a significantly increased risk of NMSC (58). Respondents diagnosed with CD are more likely to suffer from skin cancer (*p* < 0.001, 71.4 vs. 12.6%; Figure 6B).

#### 3.2.3 Dependence of skin complexion type and age on the occurrence of cancer

**Figure 7:**
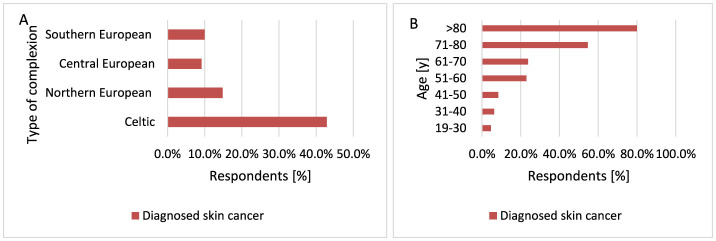
Skin cancer was diagnosed more often in participants with Celtic complexion compared to respondents with Central European complexion (*p* < 0.001) and Northern European complexion [**(A)** *p* < 0.001]. The incidence of skin cancer increased with age of participants [**(B)** *p* < 0.001].

Skin cancer is more common in older people. Mostly NMSC appears after 50 years of age. In recent years, skin cancer dramatically increased in people older than 65 years of age. Skin cancer also develops in younger people, when they have fair skin (59). Moreover, older age, male gender, Caucasian ethnicity are associated with a substantially increased risk of MM (60). Statistically, skin cancer was diagnosed more often in participants with Celtic complexion compared to respondents with Central European complexion (*p* < 0.001, 42.9 vs. 9.2%) and Northern European complexion (*p* < 0.001, 42.9 vs. 14.8%; Figure 7A). The incidence of skin cancer increased with age (*p* < 0.001, 54.6 vs. 4.7%−23.8% for 71–80 years and 80.0 vs. 4.7%−23.8% for >80 years; Figure 7B).

#### 3.2.4 Using sunscreen creams with SPF filter while staying in the sun and the using moisturizing cosmetics after a long stay in the sun or tanning

UVR is a major risk factor for developing MM, so re-protecting your skin from UVR exposure is crucial to maintaining protection against sunburn and an increased risk of future skin cancer. Sunscreens reduce the intensity of UVR acting on the epidermis, thus protecting against sunburn. Most sunscreens are chemicals that absorb various UVR wavelengths, mainly in the UVB range (1). The use of sunscreen reduces both the development of premalignant actinic keratosis and the recurrence of SCC, and at the same time, the use of sunscreen early in life may play an important role in the prevention of BCC (61).

**Figure 8:**
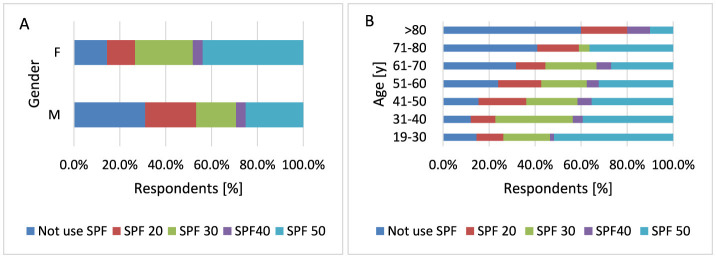
Gender **(A)** and age **(B)** differences in SPF sunscreen use: men are more likely not to use SPF sunscreen compared to women (*p* < 0.001). Women are statistically more likely to choose sunscreen with SPF50 (*p* < 0.001) and SPF30 [**(A)** *p* < 0.001]. Younger age groups, especially respondents aged 19–30 years, use SPF50 sunscreen significantly more often than other groups [**(B)** *p* < 0.001].

Men are more likely not to use SPF sunscreen compared to women (*p* < 0.001, 31.1 vs. 14.5%). If men use creams with SPF, they are more likely to choose creams with SPF20 (*p* < 0.001, 22.2 vs. 12.2%). Women are statistically more likely to choose sunscreen with SPF50 (*p* < 0.001, 43.8 vs. 25.2%) and SPF30 (*p* < 0.001, 25.2 vs. 17.4%; Figure 8A). Younger age groups, especially the group of respondents 19–30 years old, use creams with SPF50 filter statistically more often compared to other groups (*p* < 0.001, 51.7 vs 27.0%−39.2%). The 31–40 years age group of respondents uses SPF30 creams statistically significantly more often compared to other groups of respondents (*p* < 0.001, 33.5 vs. 19.8%−22.3%). In the group of older respondents over 70 years of age, they are more likely not to use SPF creams compared to younger respondents (*p* < 0.001, 40.9 vs. 12.0%−31.8% for 71–80 years) and (*p* < 0.001, 60.0 vs. 12.0%−31.8% for < 80 years; Figure 8B).

**Figure 9:**
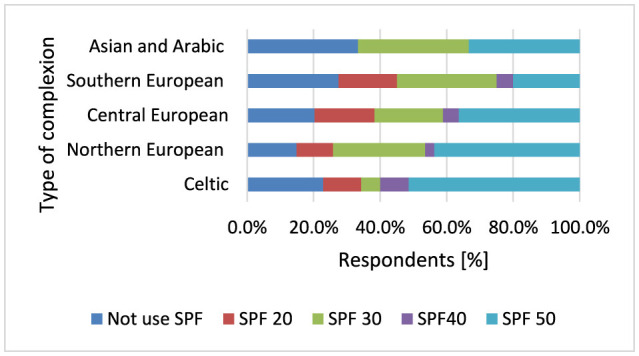
Respondents with Celtic skin type (*p* < 0.001) and Northern European skin type (*p* < 0.001) choose sunscreens with SPF50.

Respondents with Celtic skin type (*p* < 0.001, 51.4 vs. 36.4%) and Northern European skin type (*p* < 0.001, 43.8 vs. 36.4%; Figure 9) choose sunscreens with SPF50 compared to people with Central European skin type. Respondents with Southern European skin type (*p* < 0.001, 27.5 vs. 14.8%), Central European skin type (*p* < 0.001, 20.3 vs. 14.8%), Celtic skin type (*p* < 0.001, 22.9 vs. 14.8%) do not use sunscreen creams statistically more often than respondents with Northern European skin type. The rare use of sunscreens with SPF40 may be due to their lower availability, but also to the fact that dermatologists recommend using sunscreens with SPF50.

**Figure 10:**
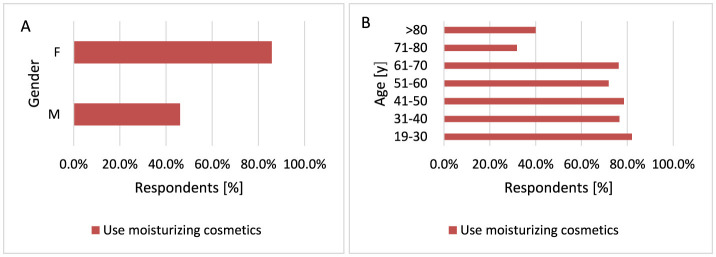
Gender **(A)** and age **(B)** differences in the use of moisturizing creams after sun exposure: women were more likely to apply moisturizing creams after prolonged sun exposure [**(A)** *p* < 0.001]. Older respondents (70+ years) were more likely not to use moisturizing creams after extended sun exposure [**(B)** *p* < 0.001].

Moisturizing prevents and alleviates skin irritation, soothing the skin by slowing the evaporation of water. Moisturizing creams are appropriate for patients with dry, sun-damaged skin (62). Women were more likely to apply moisturizing creams after longer exposure to the sun (*p* < 0.001, 85.7 vs. 46.1%) compared to men (Figure 10A). Older age groups of respondents over 70 years of age were more likely not to apply moisturizing creams after prolonged sun exposure compared to younger groups of respondents (*p* < 0.001, 68.2 vs. 18.0%−23.8% for 71–80 years and 60.0 vs. 18.0%−23.8% for < 80 years; Figure 10B).

#### 3.2.5 Using a solarium

**Figure 11:**
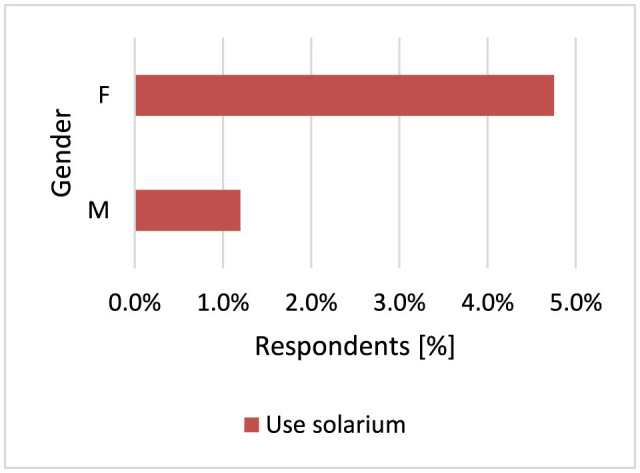
Women use solariums significantly more often (*p* = 0.039).

Tanning beds emit primarily UVA radiation, although a small amount (5%) is in the UVB range. The intensity of UVA radiation produced by large tanning units can be 10–15 times higher than that from the midday sun (1). Using a solarium is associated with a significantly increased incidence of MM diagnosed before the age of 30–40 years by over 75% (63, 64). People with fair skin are most at risk for skin cancer (61). Only 25 respondents use solariums, but it was shown that women use solariums significantly more often than men (*p* = 0.039, 4.8 vs. 1.2%; Figure 11). Of these 25 respondents, six developed skin cancer—MM [3], benign skin cancer [2] and the type of diagnosed skin cancer was unknown [1].

#### 3.2.6 Sunburn in the past

**Figure 12:**
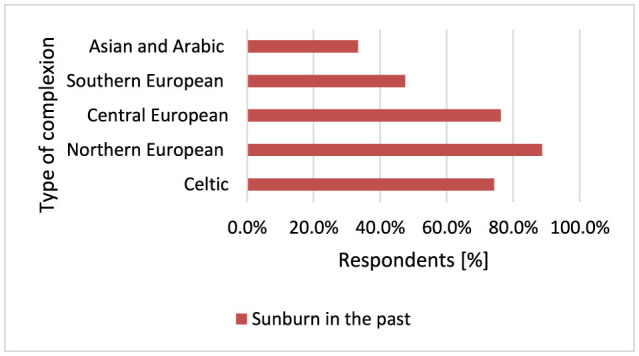
Sunburn was least common among respondents with Southern European skin (*p* < 0.001) and most common among respondents with Northern European type of complexion.

Previous sunburn may increase the likelihood of developing MM, especially at a young age (17, 65). Melanocytic nevi exposed to sunburn levels of UVR show increased melanocytic localization and cellular infiltration resembling primary MM (66). UVA rays pass deeper into the skin and can induce deeper skin damage, such as elastosis. UVB rays predominantly cause erythema or sunburn (67). A total 515 respondents had a history of sunburn. The majority of respondents (79.1%) in this study had a history of sunburn. Respondents with Southern European skin were least likely to declare having suffered sunburn compared to respondents with Northern European skin (*p* < 0.001, 47.5 vs. 88.7%), Central European (*p* < 0.001, 47.5 vs. 76.3%), and Celtic skin (*p* < 0.001, 47.5 vs. 74.3%; Figure 12).

#### 3.2.7 Wear headgear and sunglasses on sunny/hot days

**Figure 13:**
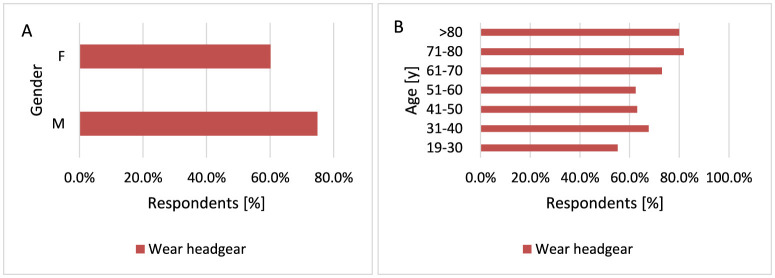
Gender **(A)** and age **(B)** differences in headgear use on sunny/hot days: men wear headgear significantly more often than women [**(A)** *p* < 0.001]. Additionally, older respondents showed a statistically significant tendency to wear headgear more frequently [**(B)** *p* = 0.040].

MM develops in parts of the body exposed to sunlight, and the frequency of melanoma lesions increases with age and the duration of exposure to UVR (17), so it is very important to ensure adequate protection of the body during exposure to UVR. Men wear headgear significantly more often than women on sunny/hot days (*p* < 0.001, 74.9 vs. 60.1%; Figure 13A). The older the respondents, the more often they wear headgear on sunny/hot days when comparing age groups over 70 years with younger groups (*p* = 0.040, 81.8 vs. 55.2%−73.0% for 71–80 years and 80.0 vs. 55.2%−73.0% for < 80 years; Figure 13B).

**Figure 14:**
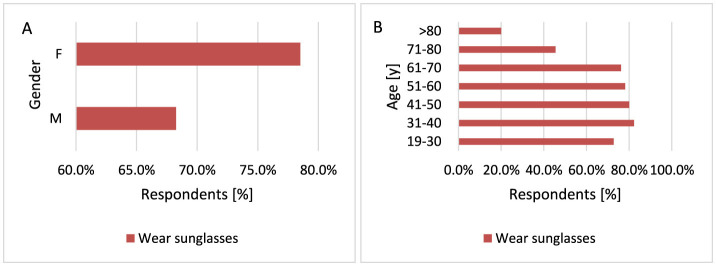
Gender **(A)** and age **(B)** differences in wearing sunglasses on sunny/hot days: women are more likely to wear sunglasses than men [**(A)** *p* = 0.018]. However, older respondents are statistically significantly less likely to wear sunglasses [**(B)** *p* < 0.001].

Respondents with higher education wear sunglasses more often compared to respondents with secondary education (*p* = 0.018, 80.4 vs. 71.8%). Women wear sunglasses significantly more often than men (*p* = 0.018, 78.5 vs. 68.3%; Figure 14A). Older participants are more likely not to wear sunglasses on sunny/hot days (*p* < 0.001, 54.6 vs. 17.7%−27.3% for 71–80 years and 80.0 vs. 17.7%−27.3% for < 80 years; Figure 14B).

#### 3.2.8 Smoking

**Figure 15:**
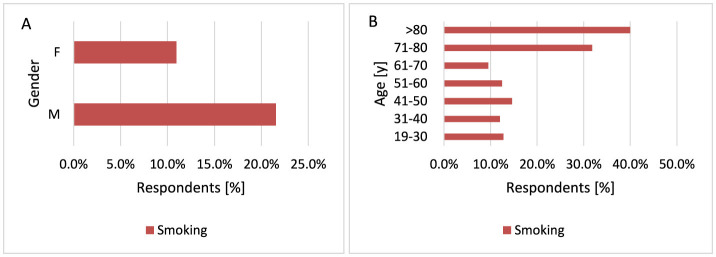
Gender **(A)** and age **(B)** differences in tobacco smoking: most respondents did not smoke tobacco, but men were significantly more likely to be smokers [**(A)** *p* = 0.006]. Additionally, the majority of tobacco smokers were over 70 years of age [**(B)** *p* = 0.034].

Tobacco smoking is a risk factor for several cancers. In a hospital-based case-control study, a relationship was demonstrated between smoking and being diagnosed and the occurrence of SCC (68). A meta-analysis of 15 studies, published between 1990 and 2018, found that current smoking was associated with higher risk of SCC but with lower risk of BCC and MM (69). The results of a cohort study suggest that patients with clinical stage I and II MM who smoked cigarettes had a significantly increased risk of death from MM (70). Most study participants do not smoke cigarettes−562 respondents. However, the study showed that men smoke tobacco more often than women (*p* = 0.006, 21.6 vs. 11.0%; Figure 15A). The majority of respondents who smoke tobacco are over 70 years of age (*p* = 0.034, 31.8 vs. 9.5%−14.6% for 71–80 years and 40.0 vs. 9.5%−14.6% for < 80 years; Figure 15B).

#### 3.2.9 Vitamin D supplementation

**Figure 16:**
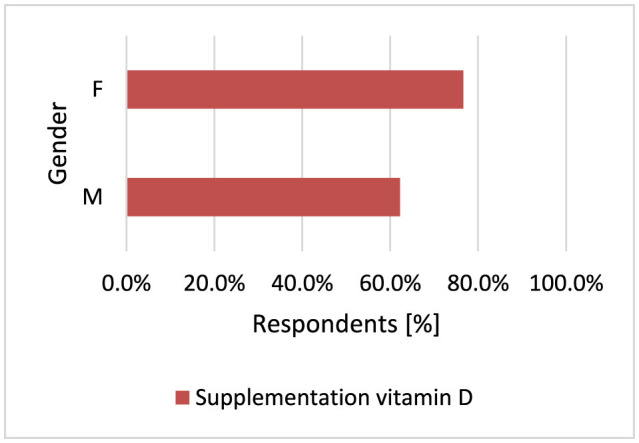
Women supplement vitamin D more often (*p* < 0.001).

The main source of vitamin D for most people is sensible sun exposure (71, 72). The vitamin D receptor has been identified in both normal melanocytes and melanoma cells (73). Several epidemiologic studies suggest that exposure to sunlight, which enhances the production of vitamin D_3_ in the skin, is important in preventing many chronic diseases (74). Both low and high levels of vitamin D are associated with an increased risk of MM (5, 23, 75). It has also been shown in an Italian case-control study that adequate dietary vitamin D reduces the risk of MM (76, 77). Vitamin D has protective effects against breast, colon, prostate cancer and even NMSC (78). Most study participants supplement vitamin D-−475 respondents. Women supplement vitamin D more often than men (*p* < 0.001, 76.7 vs. 62.3%; Figure 16).

#### 3.2.10 Diagnosis of skin cancer in a close relative (parents, siblings, and grandparents)

**Figure 17:**
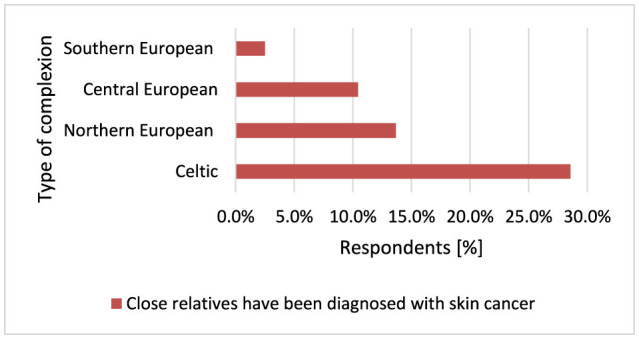
Respondents with Celtic complexion were much more likely to have close relatives diagnosed with skin cancer (*p* = 0.014).

The risk of MM increases 30–70 times in people with a significant family history of melanoma (79). There are genes whose mutations can lead to hereditary MM, such as CDKN2A and TP53 encoding protein 53 (p53) (17). Approximately 8%−10% of patients with MM have a first-degree relative with the disease. Other possible explanations for family incidence could be that the family tends to spend more time in the sun, family members share a similar skin type, or both (67). Respondents with Celtic complexion were much more likely to have people diagnosed with skin cancer in their family compared to other Central European (*p* = 0.014, 28.6 vs. 10.4%), Northern European (*p* = 0.014, 28.6 vs. 13.7%), and Southern European complexions (*p* = 0.014, 28.6 vs. 2.5%; Figure 17). Respondents with Southern European complexion very rarely had a person in their close family with skin cancer compared to people with Central European complexion (*p* = 0.014, 97.5 vs. 89.6%), Northern European (*p* = 0.014, 97.5 vs. 86.3%), and Celtic (*p* = 0.014, 97.5 vs. 71.4%).

#### 3.2.11 Mole removal procedure in the past and presence of nevi (1) Sutton's (2) Becker's (3) blue

**Figure 18:**
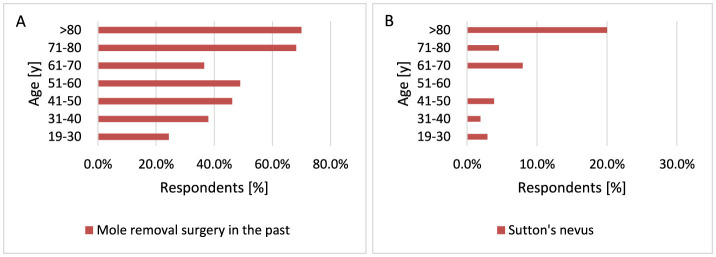
Age-related differences in mole removal **(A)** and Sutton's nevus occurrence **(B)**. Moles are removed more frequently with age (**A**, *p* < 0.001). Sutton's nevus is significantly more common in older respondents (**B**, *p* = 0.007).

The presence of multiple common or unusual moles is an accepted factor indicating an increased risk of developing MM. Benign melanocytic lesions may also act as precursors to MM (80, 81). The formation of moles is modulated by various factors, including pigmentation, genetic factors and sun exposure (7). Although pigment phenotypes and hallmarks of MM risk factors have been established, the magnitude of these associations may vary depending on geographic region. Australians have on average around three times as many moles as those living in the UK, which contributes to the higher incidence of MM in Australia (82). A total of 254 respondents had a mole removed. The study indicated that moles are removed more often with age, especially over 70 years of age compared to other age groups (*p* < 0.001, 68.2 vs. 24.2%−49.0% for 71–80 years and 70.0 vs. 24.2%−49.0% for >80 years; Figure 18A).

Melanocytic nevi are frequently accompanied by inflammatory cells of different types, in varied amounts and distributed in different patterns. Sutton's nevus is a peculiar type of regressing melanocytic nevus, also known as halo nevus (83). Sutton's nevi are found in ~1% of young adults. The most common sites for Sutton's nevi are the back, followed by head and neck (84). Clinically, the nevus is surrounded by a peripheral hypopigmented halo. The amount of the inflammatory infiltrate in halo nevus varies from moderate to dense (83). There are many diseases that have been described in individuals with Sutton's nevi, such as vitiligo, thyroid diseases, and neoplasia (84). Sutton's nevus appears significantly more often as the respondent's age increases. When comparing the 19–30 years age group with other age groups, Sutton's nevus occurs significantly more often in older respondents (*p* = 0.007, 2.9 vs. 1.9%−20.0%; Figure 18B).

**Figure 19:**
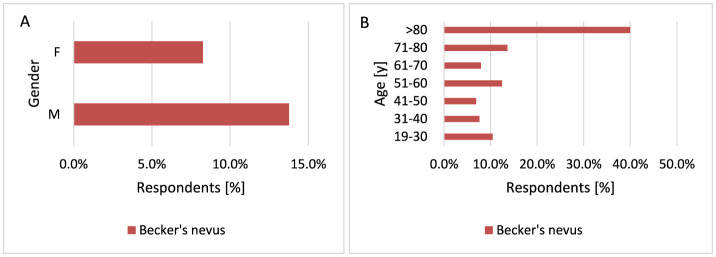
Gender **(A)** and age **(B)** differences in Becker's nevus occurrence: Becker's nevus occurs more often in men [**(A)** *p* = 0.038] and in respondents over the age of 70 [**(B)** *p* = 0.029].

Becker's nevus is a cutaneous hamartoma characterized by circumscribed hyperpigmentation with hypertrichosis. There have been reported in the literature of some patients with acneiform lesions of Becker's nevus and the hypothesis is that this lesion may be mediated by androgens (85). Becker's nevi do not pursue a malignant course but may become cosmetically problematic (86). In this study Becker's nevus occurs more often in men than in women (*p* = 0.038, 13.8 vs. 8.3%; Figure 19A). This is also confirmed by literature data, which describe the occurrence of Becker's nevus 4–6 times more often in men than in women (87). In the study population, Becker's nevus appears more often after the age of 70 (*p* = 0.029, 13.4 vs. 6.9%−12.5% for 71–80 years and 40.0 vs. 6.9%−12.5% for >80 years; Figure 19B). Most respondents did not observe the above-mentioned moles—Sutton's nevus [21], Becker's nevus [63], blue birthmark [40]. In the group of people who suffered from skin cancer—Sutton's nevus [10], Becker's nevus [17], and blue birthmark [7].

#### 3.2.12 Knowledge about the ABCDE formula for observing moles and check-ups with a dermatologist

**Figure 20:**
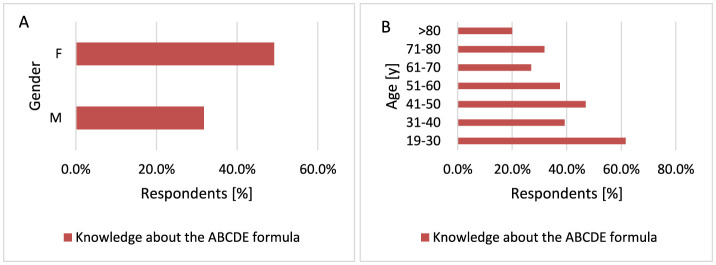
Gender **(A)** and age **(B)** differences in knowledge of the ABCDE formula: more women [**(A)** *p* < 0.001] and younger respondents [**(B)** *p* < 0.001) are familiar with the ABCDE formula.

Currently, early detection strategies for MM include teaching how to recognize suspicious lesions. The ABCDE rule describes established criteria for the occurrence of a malignant tumor by asymmetry (A), irregular borders (B), color variation (C) and diameter generally >6 mm (D), evolution (E)—in size, shape, color, surface (88, 89). More women know the ABCDE formula compared to men (*p* < 0.001, 49.2 vs. 31.7%; Figure 20A). Younger respondents know the ABCDE formula more often than older ones *p* < 0.001 when comparing the 19–30 years age group with other groups (*p* < 0.001, 61.6 vs. 20.0%−46.9%; Figure 20B). What is more, only 250 respondents (38.4%) make follow-up visits to a dermatologist.

#### 3.2.13 Knowledge about drugs causing photodermatoses and occurrence of photodermatoses after taking medications

Photodermatoses are cutaneous photosensitivity reactions that are an adverse reaction to drugs caused by exposure to sunlight (90, 91). UVR can induce an inflammatory reaction (phototoxicity) or a T-cell–mediated reaction (photoallergy). Photosensitive drugs are activated on sun exposure and undergo chemical reactions. Most photosensitive reactions are caused by UVA rather than UVB radiation (90). Not only are photosensitive reactions a cause of significant morbidity, but in some instances, pose a future risk for malignancy, specifically keratinocyte carcinoma and MM (77, 92).

**Figure 21:**
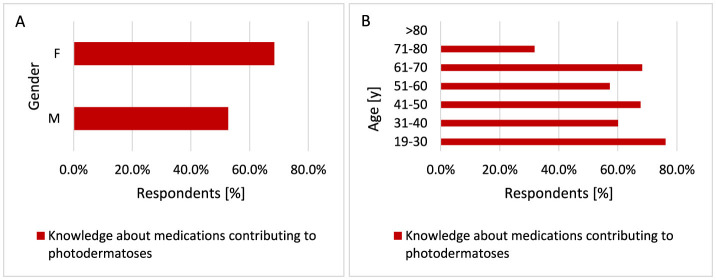
Gender **(A)** and age **(B)** differences in awareness of medications causing photodermatoses: more women [**(A)** *p* < 0.001] and younger respondents [**(B)** *p* < 0.001] are aware that using medications can lead to the occurrence of photodermatoses.

Women were more likely to know that using medications could cause the occurrence of photodermatoses compared to men (*p* < 0.001, 68.4 vs. 52.7%; Figure 21A). Respondents up to 70 years of age more often knew that drugs cause photodermatoses, especially respondents belonging to the youngest age group. Most people knew that drugs could cause photodermatoses in the 19–30 years age group compared to the other groups (*p* < 0.001, 76.2 vs. 31.8%−68.3%; Figure 21B).

**Figure 22:**
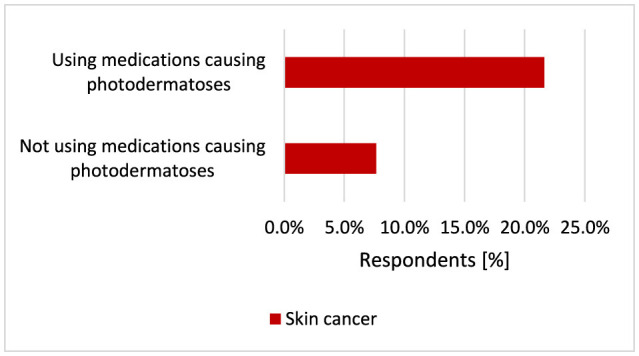
Respondents using medications that may cause photodermatoses suffered from skin cancer more often (*p* < 0.001).

Most often, photodermatoses in the group of surveyed respondents occurred after medications used for hypertension and ibuprofen, as well as contraceptives in women. Respondents using medications that may cause photodermatoses suffered from skin cancer more often (*p* < 0.001, 21.6% vs. 7.7%) (Figure 22).

**Figure 23:**
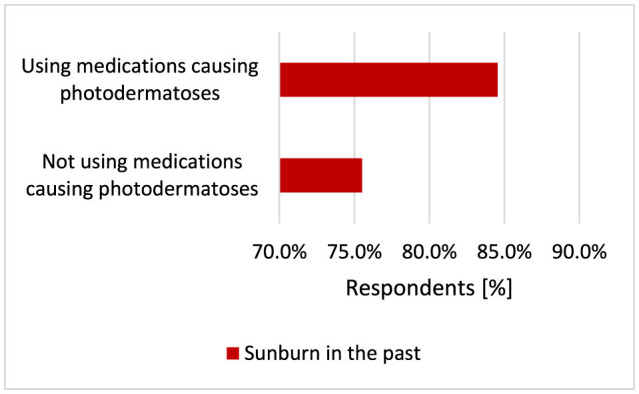
Respondents using medications causing photodermatoses more often experienced sunburn (*p* = 0.005).

**Figure 24:**
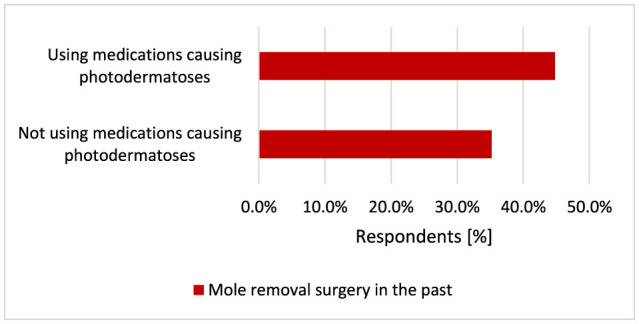
Respondents using medications causing photodermatoses more often (*p* = 0.005) had their moles removed (*p* = 0.014).

Participants using medications causing photodermatoses more often experienced sunburn statistically significantly (*p* = 0.005, 84.6 vs. 75.5%; Figure 23) and had their moles removed (*p* = 0.014, 44.8 vs. 35.2%; Figure 24).

**Figure 25:**
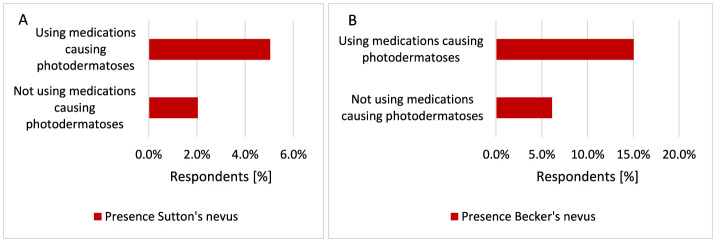
Respondents using drugs causing photodermatoses were significantly more likely to have Sutton's nevus [**(A)** *p* = 0.034] and Becker's nevus [**(B)** *p* < 0.001].

Participants using medications causing photodermatoses were significantly more likely to have Sutton's nevus (*p* = 0.034, 5.0 vs. 2.0%; Figure 25A) and Becker's nevus (*p* < 0.001, 15.1 vs. 6.1%; Figure 25B).

## 4 Discussion

The etiology of skin cancer is multifactorial, involving a complex interplay of genetic, environmental, and behavioral factors (93). The occurrence of selected skin diseases may predispose to skin cancer. Increasing evidence suggests that the increased risk of malignant tumors is associated with the occurrence of chronic inflammation, including AD (94). In this study, participants with AD were statistically significantly more likely to suffer from skin cancer (*p* < 0.001). There are conflicting data regarding the risk of skin cancer in patients with AD. A meta-analysis based on published searches in MEDLINE and Embase from 1946 and 1980, respectively, to January 3, 2019, including eight cohorts of population-based studies and 48 case-control studies, showed a statistically significant association between AD and keratinocyte carcinoma. No evidence was found of an association between AD and other cancers, including MM (16). A review of PubMed and Embase databases conducted through August 4, 2021, by another research group showed that AD is statistically significantly associated with an increased risk of BCC and SCC, but not MM (38). Similar results were obtained in a large cohort study conducted in Denmark in 1977–2006, where an inverse relationship between the co-occurrence of AD and MM was confirmed. At the same time, an increased risk of BCC and SCC has been demonstrated among people with AD (37). Also, an Italian research group found that the risk of developing BCC is increased in patients with AD, while the risk of developing MM is not increased (36). Of note, an increased risk of overall cancer was found in patients with AD compared with patients without AD (95, 96). In a case-control study conducted at United Kingdom, it was not found that patients with AD had a higher risk of developing skin cancer other than MM than other patients with dermatological diseases (97).

Atopic allergic conditions such as AD may indicate a heightened immune response, which could contribute to recognizing and removing malignant cells and thus reducing cancer risk. On the other hand, AD is accompanied by repeated tissue inflammation, damage, and repair, which could increase the risk of cancer (95, 98, 99). Mediators of the Th2 pathway also may divert tissue immunity away from an anti-tumor Th1 response (i.e., IgG1, TNF-α) and toward an IgE response against allergens, and not tumor antigens through “inappropriate Th2 immune skewing” (100). Moreover, chronic stimulation of the immune system by an antigen will induce the development of random pro-oncogenic mutations and therefore result in an increase in cancer risk. That is why, the possibility of a promoting or protective role of AD in carcinogenesis has been an interesting research area over the years (95, 98, 99). Furthermore, immunosuppressive therapies for AD such as local steroids, calcineurin inhibitors and various systemically administrated treatments (i.e., azathioprine and cyclosporine) as well as UV treatment may possibly increase the risk of cancer in general including MM (97, 101, 102).

Also, in this study participants with Ros were significantly more likely to suffer from skin cancer (*p* = 0.002). In a Denmark study an increased risk of NMSC was found among patients with Ros (40). Additionally, a cohort study in a Korean population with Ros showed an increased risk of actinic keratosis and keratinocyte carcinoma (103). In turn, the Nurses' Health Study II in the US found that the occurrence of Ros is associated with an increased risk of developing BCC (104, 105). Moreover, a German study indicated that Ros is strongly associated with MM in Caucasians (106). Several human and animal studies have shown that the most common cause of MM is cumulative exposure to UVA and UVB radiation. Exposure to UVA radiation leads to oxidative stress-induced DNA damage, and UVB induces the formation of photoproducts and the accumulation of DNA mutations. The activation of inflammatory cells such as macrophages and neutrophils during skin inflammation is associated with a malignant change in melanocytes. Due to the role of chronic inflammation and the immune system in the pathophysiology of Ros, it seems just ifiable to assume that patients diagnosed with Ros have an increased predisposition to developing MM (66, 106–110).

While the exact etiology of AA is unclear, the pathogenesis of AA is known to involve immune-mediated and inflammatory processes (111). This study showed that respondents diagnosed with AA are more likely to suffer from skin cancer (*p* < 0.001). A study from the US showed a reduced risk of developing NMSC and a trend toward a reduced risk of MM in patients with AA (112). Recent studies have demonstrated a decreased risk of MM and NMSC in vitiligo patients (113). AA has also been associated with a three- to eight-fold higher incidence of vitiligo, a skin disorder characterized by autoimmune destruction of melanocytes (114). AA and vitiligo share a similar pathogenesis, in which CD8^+^ T cells and IFN-α play an active role (112). A retrospective cohort study conducted also in the US presented a decreased risk of NMSC and a trend toward decreased risk MM in patients with AA (112). A Taiwanese study showed that the risk of NMSC was significantly lower in patients with AA (115). Also, in a study of the Korean population, the incidence of skin cancer did not increase in patients with AA (116). It is worth adding that few theories describe the potentially significant contribution of reactive oxygen species (ROS) in the pathogenesis of AA, as in AD. The results suggest that decreased antioxidant enzyme activity likely contributes to increased oxidative stress observed in patients with AA, which may indicate a common pathogenesis of AD and AA (117).

A new risk factor for the development of MM may be the occurrence of adolescent AV. A 20-year study of nurses (Nurses' Health Study II) in the US population found that women with a history of severe acne had an increased relative risk of MM. What is more, adolescents with acne were more likely to have birthmarks (46). In our study different results were obtained. Participants diagnosed with AV were significantly more likely not to suffer from skin cancer (*p* = 0.029, 5.3 vs. 14.3%). The obtained result indicating the protective effect of AV against skin diseases may be due to the fact that the respondents who took part in this survey and suffered from AV were mainly from younger age groups. The risk of skin cancer increases with age, and in this case, it is difficult to assess the impact of AV on older groups of patients suffering from skin cancer.

Current evidence also suggests that patients with psoriasis may have a higher risk of developing NMSC than patients without psoriasis (118, 119). In a Danish population study, a moderately increased risk of developing MM and NMSC was observed in patients with mild psoriasis, while in patients with severe psoriasis and psoriatic arthritis, the risk of developing NMSC was increased but did not extend to the risk of MM. Psoriasis is commonly treated with UV phototherapy and immunosuppressive drugs, which may increase the risk of skin cancer (120, 121). This study did not confirm the correlation of psoriasis in the pathogenesis of skin cancer.

DM is associated with increased prevalence of cancer including both MM and SCC (122). However, there is a lack of epidemiological data linking DM to photo-carcinogenesis (47). Genetically proxied elevated levels of HbA1c were found to be suggestively associated with a reduced risk of MM (123). Our study found that respondents diagnosed with DM have an increased risk of skin cancer (*p* < 0.001). Among men with DM, the risk of skin cancer has increased significantly in the Chinese population (124). In the Taiwanese population the incidence rate and risk of developing overall skin cancer, including NMSC, was significantly higher in older adults with DM (125). Recently, studies have also implicated vitamin D deficiency, as well as vitamin D receptor gene (FokI, BsmI, TaqI) polymorphism in the increased risk of developing both DM and MM (48).

Studies have suggested that certain glucose-lowering medications, including metformin, thiazolidinediones, insulin, and incretin-based therapies, are associated with decreased or increased risk of cancer (126). Patients using exogenous insulin had a lower risk of developing NMSC and the protective effect of insulin use becomes more distinct with increasing age (127). Also, metformin use is associated with a decreased skin cancer risk (128). A new concept in dermato-oncology is that treatment of DM and prevention of skin cancer are two sides of the same coin (122). In a Canadian population-based cohort study, glucagonlike peptide-1 receptor agonists (GLP-1 Ras) were not associated with an increased risk of NMSC or MM, compared with sulfonylureas (129). What is more, dipeptidyl peptidase 4 (DPP-4) inhibitors were associated with a reduced risk of MM but not NMSC, compared with sulfonylureas (130).

The association between hypertension and MM is unclear. This study found that hypertension increases the risk of skin cancer (*p* < 0.001). Used in therapy of hypertension hydrochlorothiazide is associated with a substantially increased risk of NMSC, especially SCC (131). In meta-analysis users of calcium channel blockers (CCB) were at increased skin cancer risk while β-blockers users were at increased risk of developing MM. There was no association between thiazide diuretics, angiotensin converting enzyme inhibitors (ACEi), angiotensin receptor blockers (ARB) use and skin cancer risk (132). A meta-analysis by a Netherlands group found that exposure to diuretics and CCB was associated with an increased risk of NMSC. This may be explained by their photosensitizing properties. Drug-induced photosensitivity indicates an adverse reaction of the skin due to the combination of sun exposure and a pharmaceutical compound. Medications in the skin may be affected by UVR, leading to the formation of ROS. This can not only lead to photo genotoxicity but also activate immune cells and the release of cytokines (133). Another meta-analysis indicated that thiazide diuretics are associated with the risk of all skin cancer types, including MM (134). Recent studies have shown a cumulative dose-dependent association between the use of hydrochlorothiazide and skin cancer, including MM and NMSC in Western Europe (135, 136).

Skin cancers were increased among treated patients with RA (137). Our study found that suffering from RA (*p* < 0.001) and CD (*p* < 0.001) increases the risk of skin cancer. The use of TNF inhibitors (138, 139) and prednisone in patients with RA was associated with an increased risk of NMSC (140). Anti-TNFs have been reported to increase the risk of MM, particularly in CD (141). Several large patient registries and clinical trial data have demonstrated the potentially causal role of immunomodulatory therapy (methotrexathe, azathioprine) in the development of skin cancer; these are also administered in CD and psoriasis (142, 143). Methotrexate-treated RA patients have an increased incidence of MM (144), and biologic therapy in RA and CD is associated with increased risk for NMSC and MM (143, 145–148).

Our study showed that, in addition to the increased risk of skin cancer in the Polish population with the coexistence of one of the diseases such as AD, Ros, AA, DM, hypertension, RA, and CD, respondents using drugs that may cause photodermatoses suffered from skin cancer more often. This confirms that, in addition to chronic inflammation in skin diseases, an important role in the development of skin cancer is played by chronic photosensitive drugs, which are prescribed for AD, Ros, AA, hypertension, RA, CD, and DM. Another result confirming that the photosensitizing drugs used may be responsible for the increased occurrence of skin cancer is that the respondents taking medications that could cause photodermatoses were more likely to suffer from skin cancer (*p* < 0.001). Furthermore, participants using drugs causing photodermatoses were significantly more likely to have Sutton's nevus (*p* = 0.034) and Becker's nevus (*p* < 0.001). Sutton's lesion should be differentiated from malignant skin tumors (83, 84). The incidence of skin cancer (*p* < 0.001), Sutton's nevi (*p* = 0.007), Becker's nevi (*p* = 0.029), and mole removal (*p* < 0.001) increased with participant age. The mean age at onset is thought to be 15 years for Sutton's nervi (84) while Becker's nevus occurs more often in men than in women (*p* = 0.038). The literature data also describe the occurrence of Becker's nevus more often in men than in women (87). Becker's nevi have been reported to have an increased amount of androgen receptors, which may explain its overall male predominance (86).

Participants using drugs causing photodermatitis statistically significantly more often experienced sunburn (*p* < 0.001) and had their moles removed (*p* < 0.001). Sunburn has been identified as a strong predictor of MM risk and has also been associated with increased risks of SCC and BCC (149–151). Among respondents suffering from skin cancer, most participants have Northern European complexion [29], which is characterized by a high tendency to sunburn, and fewer participants from this group have Celtic complexion [15], which is a very fair complexion that does not tan and immediately becomes sunburned, and Central European [29], which is a fair skin type, but is characterized by a low tendency to sunburn. Only four respondents suffering from skin cancer had a Southern European complexion. This study indicated that skin cancer was more common in people with Celtic skin (*p* < 0.001) and respondents with Celtic skin were much more likely to have family members diagnosed with skin cancer (*p* = 0.014). Skin pigmentation is one of the most important characteristics with consequences for susceptibility to skin cancer (152). In particular, of all neoplasms, ~20%−30% of skin cancers are diagnosed in Caucasians and the rate of increase of MM incidence is 3%−7% each year among Caucasians (153). Individuals with fair skin, light hair, green–blue eyes and a tendency to sunburn are at higher risk, as are those with a family history of skin cancer or genetic conditions like xeroderma pigmentosum (93, 152). Respondents with Celtic and Northern European skin types (*p* < 0.001) most often choose creams with SPF50, but respondents with Southern European skin were the least likely to declare sunburn (*p* < 0.001). It seems that protecting skin predisposed to sunburn, as in Celtic and Northern European skin types, by using sunscreen with SPF50 or not using a solarium is not sufficient to protect such individuals from skin cancer, where the genetic factor influencing the phenotype plays a dominant role in the increased risk of skin cancer. In people with skin prone to sunburn, special attention should also be paid to the controlled and judicious use of photosensitizing drugs and the need for more frequent self-observation of the skin.

The results of our survey show that the principles of protection against the development of skin cancer are observed in Polish society, which is especially justified by the fact that fair-skinned people dominate in Poland (154). Women are statistically more likely to choose creams with SPF50 (*p* < 0.001, 43.8 vs. 25.2%) and SPF30 (*p* < 0.001, 25.2 vs. 17.4%). Men are more likely not to use SPF sunscreen compared to women (*p* < 0.001, 31.1 vs. 14.5%). If men use creams with SPF, they are more likely to choose creams with SPF20 (*p* < 0.001, 22.2 vs. 12.2%). Women were more likely to apply moisturizing creams after longer exposure to the sun (*p* < 0.001, 85.7 vs. 46.1%) compared to men. On sunny days, more often men (*p* < 0.001) and older respondents (*p* = 0.040) wear headgear, and women wear sunglasses (*p* = 0.018). Women also supplemented vitamin D more often (*p* < 0.001). Most respondents do not smoke and do not use solariums. A study of the Swedish population showed that the female gender was associated with more frequent sunbathing (*p* < 0.001) and use of solariums (*p* < 0.05), but also with more frequent use of sunscreens with SPF filters (*p* < 0.001). People with low education declared using sunscreens less often than people with higher education and also chose a lower SPF (*p* < 0.001) (155).

In the German population, respondents constantly used sunscreen during holidays and while sunbathing, but much less often on a daily basis and when working outdoors. Interestingly, avoiding painful solar dermatitis was a more important motivation for respondents to use sunscreen than preventing skin cancer. The main reason for opposition to the use of sunscreen in men was the argument that applying sunscreen to the skin was too time-consuming. In the German population surveyed, the majority of respondents were also women (69%) (156) and in the Polish population surveyed (74%). Most participants in the German study had a medium or high level of education (94%) and had an even distribution of light (46%) and dark skin tones (55%) (156). In the Polish population studied, the majority of participants also had high and secondary education (94%). Most respondents in the surveyed Polish population have fair skin, prone to sunburn (participants types of complexion sensitive to sunburn: Celtic −5%, Northern European −37%, and Central European −52%). Respondents with Celtic skin type (*p* < 0.001, 51.4 vs. 36.4%) and Northern European skin type (*p* < 0.001, 43.8 vs. 36.4%) choose sunscreens with SPF50 compared to people with Central European skin type. People with fair skin, prone to burning in the sun, are at risk of developing skin cancer. Most Polish respondents have this type of complexion and clearly avoid sunbathing and willingly use sun protection products. This can be explained by the high level of awareness related to education and the desire to protect against skin cancer. The German society, however, shows great interest in sunbathing, although most respondents willingly use protective creams with SPF filter (156). Similar research results to those in the German population were obtained in a cross-sectional study of adolescents in the south of Spain (the study population consisted of 270 teenage girls). The Spanish population is characterized by a favorable attitude toward sunbathing and a tendency to use insufficient sunscreens (157). Similar results regarding attitudes toward sun protection were obtained in another German study, which assessed the impact of sunscreen use and education on the incidence of melanocytic nevi in preschool children. They found that sending educational letters and free sunscreen over a 3-year period had no additional effect on German children's sun protection (158).

In this study more women (*p* < 0.001) and younger respondents (*p* < 0.001) know the ABCDE formula for observing moles, which allows for quick identification of potential MM. Similarly, women (*p* < 0.001) and younger respondents (*p* < 0.001) are more likely to know the importance of taking medications for the occurrence of photodermatoses. Only 38.4% respondents attend follow-up visits to a dermatologist. Unfortunately, in the Polish population being diagnosed with skin cancer does not increase vigilance in skin observation and follow-up visits to a dermatologist. A retrospective cross-sectional analysis of American adults found that white women over the age of 45 with a college degree were more likely to check their skin for signs of skin cancer. Additionally, it has been shown that people with a family history of cancer were more likely to check their skin for potential skin cancer (159).

Environmental factors play an important role in the development of skin cancer, and with prevention, the risk of developing the disease can be reduced. High-profile campaigns such as the “slip, slap, slop” message (wear a T-shirt, put on a hat, slather on sunscreen) introduced in Australia have significantly raised public awareness (61). The basic strategy for preventing skin cancer involves implementing environmental, social and behavioral changes, including: using strong sunscreen and wearing protective clothing and headwears. Secondary prevention provides the opportunity to diagnose the symptoms of skin cancer and treat them at an early stage (160). Unfortunately, during the COVID-19 pandemic, MM screening campaigns were canceled due to preventive measures, which likely led to a delay in the diagnosis of skin cancers (161–163). In a retrospective study conducted at a tertiary reference center in northern Poland, data were collected on all cases of cutaneous MM treated in this facility during the official lockdown in Poland and compared with those diagnosed during the same period before the pandemic. The number of cases of cutaneous MM diagnosed during the pandemic has decreased significantly. Interestingly, this was mainly due to a decline in the number of patients with cutaneous MM located on the skin cases of trunk MM and early MM (MM *in situ* and stage pT1a) (164). In Belgium, almost 210 MM diagnoses were missed during the COVID-19 pandemic in 2020, corresponding to 6% of the expected number. This deficit occurred mainly in the first COVID-19 wave. Despite some recovery, the 2021 total was still 3% below expected, leaving ~325 diagnoses remaining to be considered in 2020 and 2021, corresponding to a 2-year period deficit at the level of 4.35% (165). A study conducted in MM treatment centers in Switzerland, Germany, Italy, and Austria showed a delay in the diagnosis of cutaneous MM due to the COVID-19 lockdown. People at high risk, such as patients with a history of MM and older people, were more likely to be hesitant to resume regular skin cancer screening after having COVID-19 (28). Surgical procedures for the diagnosis of MM and elective surgical procedures should not be postponed for longer than 3 months, therefore, public health institutions should remain functional during pandemics and offer effective solutions to build an alternative models of screening campaigns ensuring MM prevention in the conditions which are made as safe as possible within pandemic constraints (26, 166, 167).

## 5 Limitations

Limitations of this study include the following: (i) the ages of the participants are diverse, with a tendency for older people (over 70 years of age) being reluctant to participate; (ii) women are more likely to participate in the survey, while men are often reluctant to participate; (iii) as most survey respondents have secondary or higher education levels, it was not possible to assess the impact of primary or vocational education levels on factors known to offer protection from UVR.

## 6 Conclusion and recommendations

The pathogenesis of skin cancer is multifactoral. UVR in sunlight is the main etiological agent in the development of MM and NMSC. UVR produces DNA damage, gene mutations, immunosuppression, oxidative stress, and inflammatory responses, all of which play a pivotal role in photoaging of the skin and skin cancer genesis (153). The chronic inflammation, which occurs in inflammatory skin disease can damage DNA and potentially alter the risk of promoting mutagenesis, genome instability, epigenetic changes, and cytokine responses that lead to cancer (35, 135). A minority of respondents in the Polish population surveyed observe moles on the skin and make follow-up visits to a dermatologist, which makes early diagnosis of potential skin cancer lesions difficult. Moreover, limited access to healthcare resources (in terms of oncological diagnostics) caused by the fight against the COVID-19 pandemic will result in a significant number of additional deaths. Fortunately, the surveyed Polish population shows a significant interest in preventing skin cancer by using sun protection products such as creams with filters SPF, wearing headwears and sunglasses on sunny days. MM diagnosed early is completely curable, so regular examination of moles, in addition to adequate skin protection against UVR, is an important element of skin cancer prevention, especially in fair-skinned populations. In Poland, there are no campaigns raising awareness of the importance of self-observation of the skin at least once a month (using the ABCDE test) or mapping moles in a dermatologist's consulting room, which will increase the detection of skin cancer in the early stages of development. In addition, identifying people at high risk of developing skin cancer will also help optimize prevention and treatment strategies. Family doctors and clinicians should inform their patients about the increased risk of skin cancer associated with the use of some photosensitizing medicines such as β-blockers or immunosuppressants and instruct them to perform periodic skin self-examination (132).

## Data Availability

The original contributions presented in the study are included in the article/Supplementary material, further inquiries can be directed to the corresponding author.
