# Supplementary material for: Skin cancer prevention in the Polish population during the COVID-19 pandemic
Source: Front Public Health. 2025 Jun 20;13:1452043. doi: 10.3389/fpubh.2025.1452043 (PMC12226486; doi:10.3389/fpubh.2025.1452043)
Supplement: Supplementary file 1 [file Table_1.docx]

Supplementary Material

Skin cancer prevention in the Polish population during

Izabela Jęśkowiak-Kossakowska*, Jacek Calik, Adam Szeląg, Benita Wiatrak

* Correspondence: Izabela Jęśkowiak-Kossakowska

izabela.jeskowiak-kossakowska@umw.edu.pl

S1: Survey questionnaire

# The risk and course of skin cancer

The anonymous survey consists of 28 questions and will take you about 5 minutes to complete.
The survey is conducted by employees of the Department and Department of Pharmacology of the Medical University of Wrocław.

1. Education:

☐ Higher education

☐ Secondary education

☐ Professional education

☐ PhD student

☐ Student

2. Sex:

☐ Female

☐ Male

3. Age

☐ 19-30 years

☐ 31-40 years

☐ 41-50 years

☐ 51-60 years

☐ 61-70 years

☐ 71-80 years

☐ above 80 years

4. Do you have a skin disease?

☐ Atopic dermatitis

☐ Psoriasis

☐ Acne vulgaris

☐ Rosacea

☐ Vitiligo

☐ Alopecia areata

☐ Pemphigus

☐ Porphyria

☐ I am not suffer from any of the above diseases

5. Do you suffer from any disease:

☐ Ulcerative colitis

☐ Crohn's disease

☐ Hashimoto's disease

☐ Diabetes mellitus

☐ Insulin resistance

☐ Scleroderma

☐ Multiple sclerosis

☐ Hypertension

☐ Rheumatoid arthritis

☐ Cancer disease

☐ I am not suffer from any of the above diseases

6. Do you use protective creams or sunscreen when spending time in the sun?

☐ I do not use

☐ Yes, I use sunscreen with SPF 20

☐ Yes, I use sunscreen with SPF 30

☐ Yes, I use sunscreen with SPF 40

☐ Yes, I use sunscreen with SPF 50

7. Do you use a solarium?

☐ Yes

☐ No

8. Do you use moisturizing cosmetics after prolonged exposure to the sun or tanning:

☐ Yes

☐ No

9. Have you had sunburn in the past?

☐ Yes

☐ No

10. Do you wear a heatgear on sunny/hot days:

☐ Yes

☐ No

11. Do you wear sunglasses on sunny/hot days:

☐ Yes

☐ No

12. Do you visit a dermatologist for check-ups?

☐ Yes

☐ No

☐ I am undergoing skin cancer diagnosis/treatment

13. Do you smoke cigarettes?

☐ Yes

☐ No

14. Do you take vitamin D supplementation:

☐ Yes

☐ No

15. Have any of your close relatives (parents, grandparents, siblings) been diagnosed with skin cancer?

☐ Yes

☐ No

16. Have you had a mole removal procedure in the past?

☐ Yes

☐ No

17. Do you have the so-called Sutton's nevus, i.e. a birthmark surrounded by a discolored rim:

☐ Yes

☐ No

18. Do you observe the so-called Becker's nevus, an irregular, brown spot covered with hairs, located around the shoulders, arms and pelvis, which may be congenital or acquired:

☐ Yes

☐ No

19. Do you have the so-called blue birthmark, i.e. a birthmark with a blue-black or blue-gray color resembling a copy pencil, resembling an injury or a prick with a pen and ink:

☐ Yes

☐ No

20. Please choose the appropriate skin type:

☐ Celtic - very fair skin, light pink or white, blonde or red hair, light eye color (blue, gray or light green), does not tan, gets sunburn immediately

☐ Northern European - pale skin, red, light to dark blond and light brown hair, eye color blue, hazel or green, minimal tan, high tendency to burn

☐ Central European - light skin in warm tones (beige and gold), hair from dark blonde to dark deep brown, eye color grey, hazel, green or brown, always tans, slight tendency to burn

☐ Southern European - swarthy skin light brown or olive brown, hair dark brown or black, eyes intensely brown, always and easily tans, almost never burns

☐ Asian and Arabic - naturally dark olive skin, black hair, dark eyes, usually brown or black, tans well, does not tend to burn

☐ African - medium brown to dark brown skin, black hair, eye color dark brown or black, no burns

21. Do you know the so-called the ABCDE formula for mole observation, specifying the important features of a mole, the detection of which justifies reporting to a doctor. A - asymmetry, e.g. a birthmark "flowing" onto one side B - ragged, uneven edges, with thickening C - red or black and non-uniform color D - large size, size of the change: over 0.5 cm, E - evolution, i.e. progressive changes occurring in the mole:

☐ Yes

☐ No

22. Did you know that some medications may contribute photodermatoses, i.e. greater susceptibility of the skin to sunlight, which may cause burns and/or skin discoloration?

☐ Yes

☐ No

23. Do you regularly use medications that may cause photodermatoses?

☐ I do not use any of the medications listed below

☐ I take medications permanently, but I don't know which ones

☐ I only know the trade names of the drugs I take

☐ Diuretics such as hydrochlorothiazide, furosemide

☐ Drugs that lower blood pressure such as beta-blockers, which include propranolol, metoprolol, bisoprolol

☐ Antibiotics such as doxycycline, tetracycline, perfloxacin

☐ Antipsychotic phenothiazines such as chlorpromazine, promethazine, thioridazine

☐ Ketoprofen

☐ Ibuprofen

☐ Naproxen

☐ Mefenamic acid

☐ Meloxicam

☐ Oral contraceptives

☐ Anticancer medicines

24. Have you been diagnosed with skin cancer:

☐ Yes

☐ No

25. What type of skin cancer have you been diagnosed with:

☐ I do not suffer from skin cancer

☐ Skin cancer during diagnosis

☐ I don't know the type of cancer diagnosed

☐ Benign tumor (lesions are surgically removed with a small margin of healthy tissue and histopathological examination confirms the benign nature of the lesion)

☐ Melanoma

☐ Basal cell carcinoma

☐ Squamous cell carcinoma

☐ Verrucous carcinoma

☐ Merkel neuroendocrine carcinoma

☐ Cutaneous T lymphoma

☐ Paget's skin cancer

☐ Kaposi's sarcoma

26. How many years have passed since you were diagnosed with skin cancer:

☐ I do not suffer from skin cancer

☐ Up to 1 year

☐ Up to 2 years

☐ Up to 3 years

☐ Up to 4 years

☐ Up to 5 years

☐ Up to 10 years

☐ Over 10 years

27. How many years have passed since your skin cancer treatment ended:

☐ I do not suffer from skin cancer

☐ Up to 1 year

☐ Up to 2 years

☐ Up to 3 years

☐ Up to 4 years

☐ Up to 5 years

☐ Up to 10 years

☐ Over 10 years

28. Type of skin cancer treatment used:

☐ I do not suffer from skin cancer

☐ Chemotherapy

☐ Surgeon

☐ Radiotherapy

**
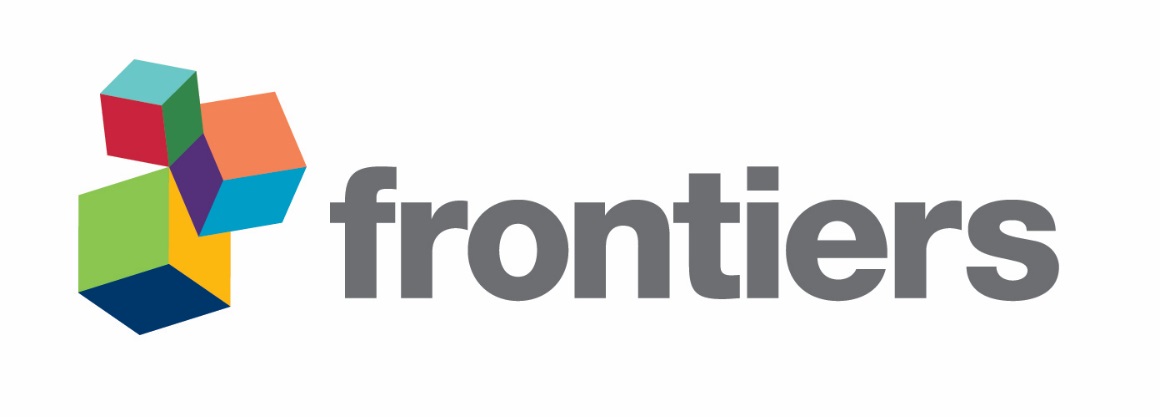
**

**Supplementary Figure 1.** The figure legends are required to have the same font as the main text, 12 point normal Times New Roman, single spaced. Please use a single paragraph for each legend and prepare the figures keeping in mind the PDF layout.
